# Analyses of Lysin-motif Receptor-like Kinase (*LysM-RLK*) Gene Family in Allotetraploid *Brassica napus* L. and Its Progenitor Species: An In Silico Study

**DOI:** 10.3390/cells11010037

**Published:** 2021-12-23

**Authors:** Amin Abedi, Zahra Hajiahmadi, Mojtaba Kordrostami, Qassim Esmaeel, Cédric Jacquard

**Affiliations:** 1 Department of Biotechnology, Faculty of Agricultural Sciences, University of Guilan, Rasht 41635-1314, Iran; abedi.amin@yahoo.com (A.A.); z.hajiahmadi1366@gmail.com (Z.H.); 2 Nuclear Agriculture Research School, Nuclear Science and Technology Research Institute (NSTRI), Atomic Energy Organization of Iran (AEOI), Karaj 31485-498, Iran; 3 Université de Reims Champagne-Ardenne, RIBP EA4707 USC INRAE 1488, SFR Condorcet FR CNRS 3417, 51100 Reims, France; qassim.esmaeel@univ-reims.fr

**Keywords:** bioinformatics, codon usage bias, expression pattern, in silico study, modeling, molecular docking

## Abstract

The LysM receptor-like kinases (LysM-RLKs) play a crucial role in plant symbiosis and response to environmental stresses. *Brassica napus*, *B. rapa*, and *B. oleracea* are utilized as valuable vegetables. Different biotic and abiotic stressors affect these crops, resulting in yield losses. Therefore, genome-wide analysis of the LysM-RLK gene family was conducted. From the genome of the examined species, 33 LysM-RLK have been found. The conserved domains of *Brassica* LysM-RLKs were divided into three groups: LYK, LYP, and LysMn. In the *Brassica* *LysM-RLK* gene family, only segmental duplication has occurred. The Ka/Ks ratio for the duplicated pair of genes was less than one indicating that the genes’ function had not changed over time. The *Brassica* *LysM-RLKs* contain 70 cis-elements, indicating that they are involved in stress response. 39 miRNA molecules were responsible for the post-transcriptional regulation of 12 *Brassica LysM-RLKs*. A total of 22 SSR loci were discovered in 16 *Brassica LysM-RLKs*. According to RNA-seq data, the highest expression in response to biotic stresses was related to BnLYP6. According to the docking simulations, several residues in the active sites of BnLYP6 are in direct contact with the docked chitin and could be useful in future studies to develop pathogen-resistant *B. napus*. This research reveals comprehensive information that could lead to the identification of potential genes for *Brassica* species genetic manipulation.

## 1. Introduction

Because plants are stationary, they are subjected to a variety of biotic and abiotic stresses throughout their lives. Plants have been developed their immune strategies using signal transduction from the site of infection [1]. Immune receptors are used by plants to detect and respond to invading pathogens [2]. Plants’ immune receptors are classified as either nucleotide-binding leucine-rich repeat receptors (NLR) or pattern recognition receptors (PRRs) [3]. Accordingly, NLR and PRR receptors are known as resistance gene analogs (RGAs). NLRs play a major role in plant disease resistance and are also known as resistance genes (R genes) [4].

PRRs are the main line of defense against infections. They are located on the cell membrane and belong to a receptor kinase family. They contain an intracellular kinase domain, a transmembrane domain, as well as an extracellular ligand-binding domain [5]. The extracellular domain recognizes molecular-associated molecular patterns (MAMPs). MAMPs are cell envelope components such as lipopolysaccharide (LPS), flagellin, chitin, β-glucans, peptidoglycan, and ergosterols [2,6]. Proteins containing Lysin-motif (LysM) are PRRs that recognize MAMPs [3,7]. Plant PRRs are classified into two groups: receptor-like proteins (RLPs) and receptor-like kinases (RLKs). RLKs contain an intracellular kinase domain, a transmembrane domain, and also an extracellular ligand-binding domain that is involved in signal transduction while RLPs lack intracellular regions [8]. Plant RLKs have a similar structure to animal receptor tyrosine kinases. Several extracellular domains have been discovered in plants, but none have been identified in animals [9]. In their extracellular regions, plant RLKs exhibit a diverse set of domains [10]. Plant RLKs can be divided into 14 groups, which include wall-associated kinase (WAK), receptor-like kinase in flowers (RKF), CRINKLY-like (CR-like), *Catharanthus roseus* like (CrRLK), the domain of unknown function 26 (DUF26), lectin (C-Lectin and L-Lectin), leucine-rich repeat (LRR), lysin motif (LysM), an extension like proline-rich extensin like (PERK), leaf rust kinase-like (LRK), thaumatin, self-incompatibility domain (S-domain), and unknown receptor kinase (URK) with various functions [9]. Few RLKs are known to have crucial roles in plant defense such as lysin motif- receptor-like kinases (LysM-RLKs), one of the most important groups implicated in plant defense response. LysM has a highly conserved βααβ secondary structure containing about 50 amino acids that bind to chitin and peptidoglycans [11]. It was first discovered in lysozyme [12] of a bacteriophage and later shown to be present in a wide range of eukaryotes and prokaryotes [13,14]. LysM family contains LysM-containing receptor-like kinase (LYK) and LysM-containing receptor-like proteins (LYP) which are widely distributed in the plant kingdom [15,16]. LYKs are important in plant-pathogen interactions because they activate the immune system by sensing pathogen entrance into the host cell [17]. The LysM motif is found in the extracellular region of LYKs [18]. LYKs are essential mediators of innate immunity against pathogens in a broad range of plant species. Investigation of the properties of the plant LysM showed that plant genomes have at least 11 distinct types of this motif, which are highly diverse in plants and at least six types of LysM motif in LysM kinase proteins and five other types in non-kinase LysM proteins have been identified [19]. The first receptor protein (*Oryza sativa* chitin elicitor-binding protein- *OsCEBiP*) was identified in rice which contains two extracellular LysM domains and a transmembrane LysM domain. Silencing of *OsCEBiP* prevented the plant from responding to chitin [15]. Wheat and barley have orthologs of *OsCEBIP* that are engaged in the plant defence system. The *Mycosphaerella graminicola* pathogen caused disease symptoms in wheat lines that were knocked down for *TaCEBIP* (*Triticum aestivum* chitin elicitor binding protein) using the virus-induced gene silencing (VIGS) approach [20]. In the barley lines knocked down for *HvCEBIP* (*Hordeum vulgar* chitin elicitor binding protein) via the VIGS, increased lesions owing to *Magnaporthe oryzae* infection were also observed [21]. Similarly, OsCERK1 (*Oryza sativa* chitin elicitor receptor kinase 1) is a LysM receptor-like protein kinase with three lysine motifs and a kinase domain that is required for signal transduction in rice. However, OsCEBiP is the main chitin-binding protein that uses OsCERK1 to activate the chitin-stimulated immune response [22,23,24]. OsCERK1 transmits the chitin signal to the cell, which has been sensed by OsCEBiP. CERK1 directly binds to chitin by three extracellular LysM domains without any other proteins indicating that it is the main chitin-binding protein in plants [25]. The activation of Mitogen-activated protein kinase (MAPK), the formation of reactive oxygen species (ROS), and the expression of defense genes are all part of the immunological response caused by fungal chitin [26]. According to certain studies, AtCERK (*Arabidopsis thaliana* chitin elicitor receptor kinase) has a dual role in biotic and abiotic stress signaling [27]. In Arabidopsis, *Atlyk1* and *Atlyk4* bind to chitin and transmit extracellular signals to the cell, activating downstream pathways of disease resistance [28]. AtLYM1 and AtLYM3 are similar to OsCEBiP and have a role in binding to peptidoglycans [29]. AtLYM2 has been reported to increase plant resistance against *Botrytis cinerea* and *Alternaria brassicicola* [30,31]. VvLYK1-1 and VvLYK1-2 in grapevine are involved in immunity induced by chitin and chitosan, and hence VvLYK1-1 is implicated in *Erysiphe necator* resistance [32].

In addition to the plant immune response, LysM-RLK genes are also involved in the plant and arbuscular mycorrhizal (AM) fungi interaction. Studies on chickpeas have shown that PsLYK9 is directly involved in the perception of long- and short-chain oligosaccharides in the hydrolyzed cell walls of the fungus and plays an important role in the immune response of chickpeas to fungal pathogens. PsLYK9, on the other hand, is involved in the symbiosis development of AM fungi, so that silencing *PsLYK9* reduces levels of colonization by mycorrhizal fungi [33]. In tomatoes, SlLYK1 and SlLYK13 are involved in the chitin-induced immune response and cell death, respectively. While SlLYK10 and SlLYK13 participate in the regulation of AM symbiosis [34,35].

*Brassica napus* L. (2n = 4x = 38) is one of the most important allopolyploid oilseed crops derived from the hybridization of *B. oleracea* (2n = 2x = 18), and *B. rapa* (2n = 2x = 20). These species are used as important vegetables in the form of sauces, oil, and fodder, among many other items. Their crops are subjected to biotic and abiotic stressors throughout their life cycle, resulting in yield reductions. *Alternaria* spp., *Fusarium oxysporum*, *Albuga candia*, and *Leptosphaeria maculans* are the most prevalent fungal diseases that affect *Brassica* [36]. *Brassica* crops are susceptible to bacterial rot disease as well [37]. Similarly, salinity and drought among abiotic stresses have the highest impact on *Brassica* yield [38]. To boost crop productivity, stress-tolerant *Brassica* can be developed through genetic engineering. In Arabidopsis, rice, lotus, sweet orange, potato, wheat, Chinese white pear, as well as other crops, the Lysin-Motif Receptor-Like Kinase (LysM-RLK) family has been explored [8,17,39,40,41,42,43,44]. However, no genome-wide association studies in *B. napus* have been conducted to date. As a result, functional investigations of *LysM-RLKs* in *Brassica* have been investigated using bioinformatics techniques, given the importance of *Brassica* and the decrease of its products owing to biotic and abiotic stressors.

## 2. Materials and Methods

### 2.1. In Silico Identification of LysM-RLK Genes

The HMM profile of the LysM domain (Pfam ID: PF01476) was retrieved from the Pfam database [45], and the HmmerSearch tool [46] was used to determine *Brassica* LysM-RLK proteins in the Ensembl Plants database to determine the LysM-RLK gene family in *B. napus*, *B. oleracea*, and *B. rapa*. Three domains including F-box-like (PF12937), protein tyrosine kinase (PF07714), and protein kinase domain (PF00069) were identified. The default parameters include significance E-values of 0.01 for sequence and 0.03 for hit matches, as well as reporting E-values of 1 for both sequences and hit. The ProtParam tool of the ExPASY bioinformatics resource portal [47] was used to compute the molecular weight, length, and theoretical isoelectric points of *Brassica* LysM-RLK. CELLO and DeepLoc were used to predict protein cellular localization [48,49].

### 2.2. Phylogenetic Relationships of Brassica LysM-RLK Gene Family

To study the evolutionary relationships of the *LysM-RLK* gene family, full-length protein sequence alignments of *B. napus* (Bn), *B. oleracea* (Bo), *B. rapa* (Br), *Arabidopsis thaliana* (At), *Brachypodium distachyon* (Bd), *Oryza sativa* (Os), and *Vitis vinifera* (Vv) were performed using ClustalX 2.0.8 software. The Neighbor-joining (NJ) method with 1000 bootstraps was used to generate a phylogenetic tree of LysM-RLK proteins using MEGA 7 [50] and the p-distance model [51].

### 2.3. Investigation of Chromosome Localization, Gene Duplication, and Selection Pressure of LysM-RLK Members

The coding sequence (CDS) of the examined genes was retrieved from the Ensemble Plants database using the biomart program [52] to investigate duplication and selection pressure. Tandem duplication is defined as the duplication of genes on the same chromosome separated by no more than 10 genes [53]. Similarly, two criteria were utilized to detect segmental duplication: the aligned region’s identity had to be larger than 90% and the alignment coverage had to be greater than 90% [54]. Synonymous (Ks) and non-synonymous (Ka) substitution rates were evaluated using DnaSP ver. 5 software [55] to establish the kind of selection pressure. TBtools [56] was used to determine the location of genes on chromosomes and the duplication relationship among them.

### 2.4. Exon-Intron Structure and Conserved Motifs of BLysM-RLK

The Multiple Em for Motif Elicitation (MEME 5.0.5) algorithm was used to find specific *LysM-RLK* gene motifs [57]. Twenty motifs with a minimum and maximum length of motifs 6 and two hundred amino acids have been considered. These findings were shown using the TBtools software [56]. The GFF3 file linked to the three *Brassica* species was retrieved from the Ensemble Plants database and the appropriate analyses were done using TBtools software [56] to illustrate the exon-intron structure of the examined genes.

### 2.5. The Prediction of Cis-Regulatory Elements, Simple Sequence Repeats (SSR) Markers, and BLysM-RLK-Targeted miRNAs

PlantCare [58] was used to identify cis-acting regulatory elements of 1500 bp upstream of the initiation codon (ATG) of the *LysM-RLK* genes from the Ensemble Plants database [52]. SSR markers in BLysM-RLK genes were discovered using the BatchPrimer3v1.0 server [59]. CD sequences of them were evaluated in the psRNATarget database using default parameters to detect in *BLysM-RLK*-targeted miRNAs.

### 2.6. Codon Usage Bias Analysis

CodonW 1.4.2 was used to analyze the sequences for frequency of optimal codons (FOP), codon adaptation index (CAI), GC content, effective codon number (ENC), GC content at the third site position of a codon (GC3s), and relative synonymous codon usage (RSCU) for *Brassica* LysM-RLK [60]. The statistical analysis was carried out using Excel software.

### 2.7. RNA-Seq Analysis of Brassica LysM-RLK Genes

The transcript data for flower, leaf, root, silique, and stem tissues as well as dehydration stress at 1 and 8 h after treatment and ABA (25 M), cold (4 °C), and salinity (200 mM), stresses at 4 and 24 h after treatment were related to the study of Zhang et al. [61] with the project ID CRA001775 [62]. FastQC software [48] was used for the initial quality analysis on FastQ files, and then the raw sequence data was preprocessed and adapter sequences, low-quality reads, and duplicate mapping reads were filtered using Trimmomatic on Linux [49]. The preprocessed FastQ files were aligned to the *Brassica napus* reference genome using STAR [50]. The counts obtained from STAR normalized to transcript per million (TPM). Log2 (TPM + 1) used to generate the heatmap utilizing TBtools [63]. Clustering the data was performed using the Pearson correlation coincident and the complete linkage method. Similarly, the BrassicaEDB database was used to study the expression of *BnLysM-RLK* genes in response to fungal infections such as *Leptosphaeria maculans* and *Sclerotinia sclerotiorum*. Expression data related to the *Leptosphaeria Maculans* inoculation is available in the NCBI with the project ID number PRJNA311316. In total, they sequenced 36 samples (18 from the resistant (LepR1) genotype and 18 from the susceptible genotype (Westar)). Samples were collected 0, 3, 7, and 11 days post-inoculation in triplicate. RNA-seq data with accession number PRJNA274853 publicly available on the NCBI SRA database were mined and analyzed for expression patterns of the rapeseed *LysM-RLK* genes in response to S. sclerotiorum infection. The experiment consisted of 24 samples containing susceptible (J902) and resistant (J964) genotypes and was sampled at 24, 48, and 96 h after treatment with three biological replications.

### 2.8. Structural Modeling and Validation

Iterative template-based fragment assembly simulations were used to create the full-length atomic structures of BnLYP6 proteins to forecast protein structures on the I-TASSER server [64]. The top models from I-TASSER were refined using the ModRefinder software [65]. Ramachandran plot has been applied to confirm the predicted structures by measuring the backbone dihedral phi (ϕ) and psi (Ψ) angles with the PROCHECK module of the PDBSum server [66].

### 2.9. Molecular Docking

The chitin ligand structure was retrieved from the PubChem database [67] and converted to PDB format using Discovery Studio software. An improved version of the COACH server (COACH-D) was utilized to discover protein-ligand interaction sites [68]. To suggest protein-ligand binding sites, the aforementioned server employs five approaches, four of which are template-based, including TM-SITE [69], COFACTOR [70], and FINDSITE [71] while the last method (ConCavity) is based on structure [72]. The results of each approach were then combined using the COACH algorithm [69]. The ligand-enzyme interaction was studied using AutoDock v4.2.6 [73]. The Auto Grid application, which was created with AutoDock, was used to create grid maps. The grid box sizes for x, y, and z were set to 82, 90, and 120, respectively. The grid centers for x, y, and z were set at 73.866, 76.789, and 68.402, respectively, with a grid spacing of 0.375. To find the best conformers, the Lamarckian Genetic Algorithm (LGA) was used. During the docking process, a limit of 200 conformers was considered for the ligand. The default AutoDock4 parameters were used for the majority of docking processes [73]. The maximum number of tests was set at 2,500,000, the population size was set at 150, the maximum number of generations was set at 27,000, the maximum number of automatically surviving top individuals was set at 1, the gene mutation rate was set at 0.02 and the crossover rate was set at 0.8. The interaction of enzymes and substrates has been illustrated in 2D and 3D using Discovery Studio Visualizer and Chimera softwares [74].

## 3. Results

### 3.1. Identification of Brassica LysM-RLK Genes

In the current investigation, 33 *LysM-RLK* genes were discovered (17 in *B. napus*, 8 in each of *B. rapa* and *B. oleracea*). The prefix Bn, Bo, and Br, as well as the protected domain discovered in each gene, were used to label the identified *LysM-RLK* genes. The chromosomal location of the genes has been used to estimate the gene number. They were divided into three groups based on their specific domains including LYK (5 in *B. napus*, 2 in each of *B. rapa* and *B. oleracea*), LYP (10 in *B. napus*, 5 in each of *B. rapa* and *B. oleracea*), and LysMn (2 in *B. napus*, 1 in each of *B. rapa* and *B. oleracea*) (Table 1). LYKs are made up of LysM and protein kinase domains, according to supplementary file 1 (Table S1). LysM domains have been discovered in LYPs. Some LYPs are transmembrane, whereas others use a glycosylphosphatidylinositol anchor to bind to the membrane. LysMn proteins have an F box-like domain with extracellular or plasma membrane localization. The physicochemical characteristics of LysM-RLK were investigated using the ProtParam tool. The length of these 33 BnATGs protein sequences ranged from 260 amino acids (BnLysMn1-2, BrLysMn, and BoLysMn) to 665 amino acids (BnLysMn1-2, BrLysMn, and BoLysMn) (BnLYK3 and BoLYK1). The molecular weights of LysM-RLK proteins ranged from 4.08 to 72.76 kDa, with isoelectric points (*pI*) ranging from 4.64 to 7.78 (Table 1). Based on the *pI* value, the majority of proteins (28 members, 84.84%) were acidic.

### 3.2. Phylogenetic Analysis of LysM-RLK Proteins

**Figure 1:**
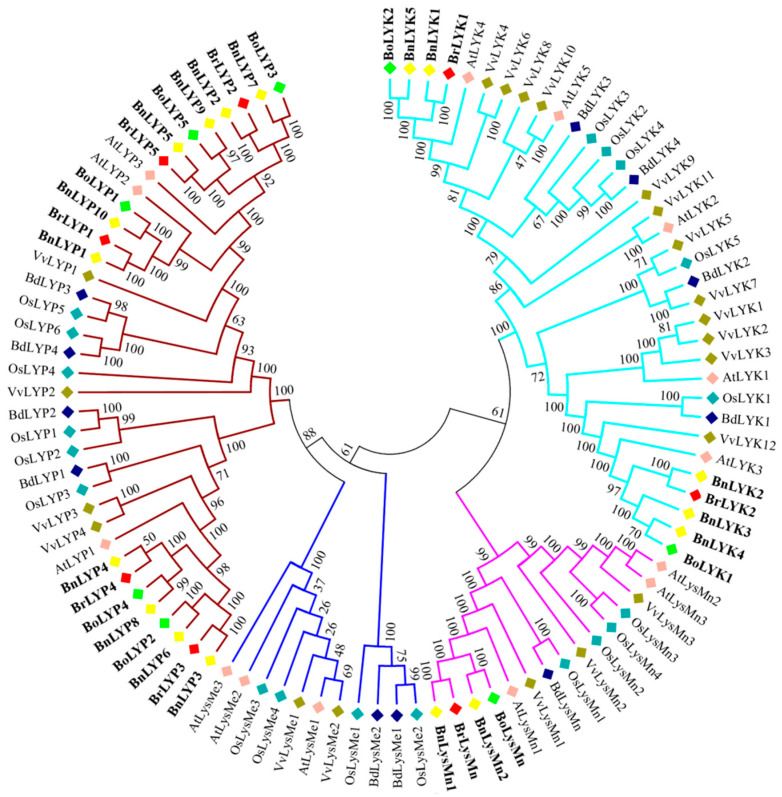
Phylogenetic relationships of *LysM-RLK* genes from *Brassica napus* (Bn), *Brassica rapa* (Br), *Brassica oleracea* (Bo), *Oryza sativa* (Os), *Arabidopsis thaliana* (At), *Vitis vinifera* (Vv), and *Brachypodium distachyon* (Bd). Colored branches have been used to depict various subfamilies. The phylogenetic dendrogram was constructed using MEGA 7 software and the neighbor-joining (NJ) method with 1000 bootstraps.

A neighbor-joining phylogenetic dendrogram was constructed to establish the link between *Brassica* LysM-RLK proteins and their homologous in other plants. According to Figure 1, the LysM-RLKs in *Brassica* were highly similar to their counterparts in Arabidopsis (At), rice (Os), and grapevine (Vv). LysM-RLK proteins were divided into four subfamilies: LYK, LYP, LysMe, and LysMn. Except for LysMn, all subfamilies have been identified in the three *Brassica* species investigated. Based on previous studies in Arabidopsis, AtLYP1 and AtLYP3 recognize peptidoglycan while AtCERK1, AtLYK4, and AtLYK5 recognize chitin. Therefore, due to the existence of the BnLYP2, BrLYP2, BoLYP2, BnLYP3, BrLYP3, BoLYP3, BnLYP4, BrLYP4, BoLYP4, BnLYP5, BrLYP5, BoLYP5, BnLYP6, BoLYP7, BnLYP8, BnLYP9 in the clade of AtLYP1 and AtLYP3, they can recognize peptidoglycan while BnLYK1, BrLYK1, BnLYK5, and BoLYK2 formed a monophyletic cluster with AtLYK4 confirming their ability to recognize chitin. It seems that LYP and LYK subgroups can specifically identify peptidoglycan and chitin, respectively. However, some studies reported that some members of the LYP subfamily can bind to both chitin and peptidoglycan ligands such as LYP1, LYP4, LYP5, and LYP6 [25,63,75,76].

### 3.3. Gene Duplication, Gene Location on the Chromosomes, and Selection Pressure of LysM-RLK Genes

**Figure 2:**
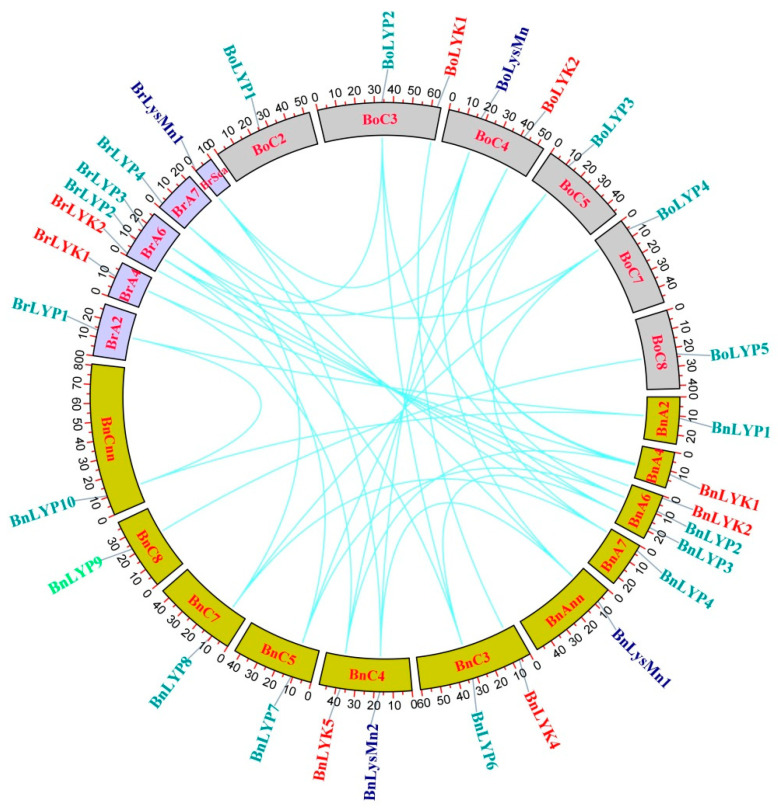
The chromosomal location of *LysM-RLK* genes and the duplication relationship between them. Colored boxes represent chromosomes. Curves are used to show gene duplications.

The chromosomal distribution of 33 *Brassica LysM-RLK* was unequal on chromosomes (Figure 2). Chromosome (Chr) A6 in *B. rapa* and *B. napus* had the most genes, while ChrC3 and ChrC4 revealed the highest number of genes in *B. oleracea*. The genes of each subfamily were located on different chromosomes. In LYP subfamily of *B. napus*, *LYP1, LYP2-3, LYP4, LYP5, LYP6, LYP7, LYP8, LYP9*, and *LYP10* were found on ChrA2, ChrA6, ChrA7, ChrA8, ChrC3, ChrC5, ChrC7, and ChrC8, respectively. *BnLysMn1* and *BnLysMn2* were identified on ChrAnn and ChrC4, respectively, in the LysMn subfamily of *B. napus*. Only segmental duplication was found in the *Brassica LysM-RLK* gene family, according to duplication analyses (Supplementary Materials: Table S2). Gene duplication is an effective phenomenon contributing to the abundance of duplicate genes in plant genomes which have contributed to the evolution of novel functions. To indicate selection pressure between duplicated genes, the Ks, Ka, and Ka/Ks parameters were investigated for 43 paired genes (Supplementary Materials: Table S3).

Except for *BnLysMn2/BnLysMn1*, *BnLysMn2/BrLysMn*, and *BnLYP6/BrLYP3*, the Ka/Ks ratio of 43 paired genes was less than 1, showing negative selection to maintain their function during *Brassica* evolution. The Ka/Ks ratio for three paired genes (*BnLysMn2/BnLysMn1*, *BnLysMn2/BrLysMn*, and *BnLYP6/BrLYP3*) was more than one, indicating positive selection, which resulted in their various functions as a result of mutations during their evolution.

### 3.4. Exon-Intron Structures and Conserved Motifs of Brassica LysM-RLKs

**Figure 3:**
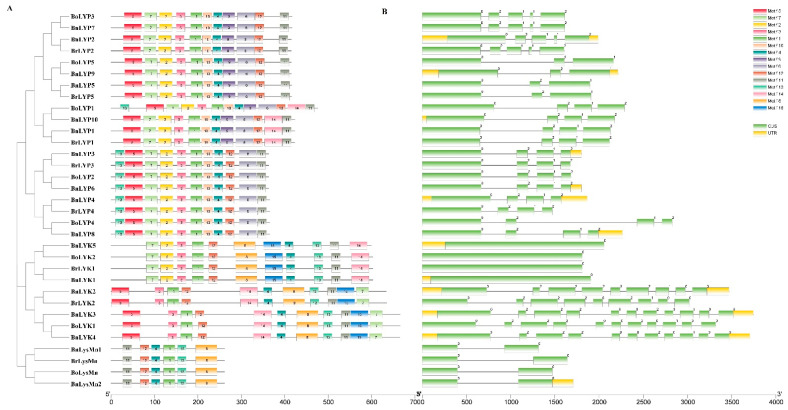
The conserved motifs (**A**) and exon-intron structure (**B**) of *LysM-RLK* genes in *Brassica* species. Exons and introns were represented by green boxes and black lines, respectively. Different motifs are shown by different colors Exon-intron structure and Motifs were determined using gene structure display server (GSDS) and MEME online tool, respectively.

The MEME tool was used to find conserved motifs in *Brassica* LysM-RLK protein sequences (Supplementary Materials: Table S3). According to the data, 15 conserved motifs have been discovered, although the lowest number of motifs was detected in LysMn with 6 motifs (Figure 3A). The highest number of motifs was related to the BoLYP1 with 13 motifs, followed by BnLYP1, BrLYP1, and BnLYP10 with 12 motifs. As expected, each subgroup showed approximately similar motif compositions. *Brassica LysM-RLK* contains 0 to 10 introns, with *BoLYP4* being the longest intron, according to the exon-intron structural study (Figure 3B). Intron-free *Brassica LysM-RLK* genes account for 9.09% of the genome. The majority of *Brassica LysM-RLK* genes exhibited zero, one, or two forms of intron splicing, but *BnLYK5*, *BoLYK2*, *BoLYK1*, *BrLYK1*, *BnLysMn1*, *BnLysMn2*, *BoLysMn*, and *BrLysMn* had intron phase splicing zero. Exons ranged from one to five in *Brassica LysM-RLKs*, whereas *BnLYK2, BnLYK4, BnLYK3, BrLYK2,* and *BoLYK1* contained nine and eleven exons, respectively. The highest amount of diversity in the number of exons was observed in the LYK subfamily, which indicates a selective pressure to obtain different functions during the evolution of *Brassica* [77]. Each subfamily showed similar intron splicing phases. The LysMn subfamily only displayed splicing phase zero, whereas the LYP subfamily showed all three splicing phases. Based on the splicing phase, the LYK subfamily was separated into two groups: (1) *BnLYK5*, *BoLYK2*, *BoLYK1*, and *BrLYK1* with splicing phase zero, and (2) *BoLYK1*, *BnLYK2*, *BrLYK2*, *BnLYK3*, and *BnLYK4* with all three splicing phases. The untranslated region was only found in 10 of the *Brassica LysM-RLKs* including *BnLYP2-3*, *BnLYp4*, *BnLYP6*, *BnLYP8-10*, *BnLYK1-5*, and *BnLysMn2*.

### 3.5. The Prediction of Cis-Regulatory Elements, Simple Sequence Repeats (SSR) Markers, and Brassica LysM-RLK-Targeted miRNAs

PlantCare was used to detect cis-regulatory elements in 1500 bp upstream of the *Brassica* LysM-RLK start codon (Supplementary Materials: Table S4). The *Brassica LysM-RLK* gene family has been discovered to have 70 cis-elements that can control gene expression in response to five different factors: environmental stresses, light, circadian, phytohormones, and developmental stages. The highest frequency of cis-acting elements in *B. napus*, *B. oleracea,* and *B. rapa* was related to ARE (94.11%), MYC (100%), and ARE (100%), respectively. The lowest frequency of cis-regulatory elements was related to GC-motif (only in *BoLYP2*) AT-rich sequence (only in *BnLYK4*), CARE (only in *BnLYK2*), GTGGC-motif (only in *BnLYR1*), MSA-like (only in *BoLYR2*), and F-box (only in *BnLYP5*). *Brassica* LysM-RLK contained 218 stress-responsive elements, indicating that they may have a role in regulating the Brassica response to different environmental challenges. 168, 161, and 75 cis-acting elements associated with phytohormones, light, and different tissues were also detected. Therefore, *Brassica* LysM-RLKs have the potential to play a role in a variety of processes. 22 SSRs were identified in 16 out of 33 *Brassica* LysM-RLKs (13 SSRs in *B. napus*, 5 SSRs in *B. rapa*, and 4 SSRs in *B. oleracea*) (Table 2). Most genes had a single SSR except *BnLYP5* (2 SSRs), BnLYP2, and *BnLYP9* (4 SSRs each). The highest frequency was related to tetra-nucleotide repeats (9 SSRs) followed by di-nucleotide repeats (6 SSRs), tri-nucleotide repeats (4 SSRs), and pentanucleotide repeats (3 SSRs). 39 miRNAs for 12 *Brassica LysM-RLKs* targets have been detected (Supplementary Materials: Table S5). miRNAs and their targets did not have a one-to-one relationship, and many miRNAs shared a common target. For instance, 10 miRNAs named bra-miR156a-5p, bra-miR156b-5p, bra-miR156c-5p, bra-miR156d-5p, bra-miR156e-5p, bra-miR156f-5p, bra-miR156g-5p, bra-miR5725, bra-miR5721, and bra-miR9565-3p co-targeted *BrLYP2* transcript. One miRNA such as bna-miR390a can suppress the expression of multiple targets including *BnLYK1*, *BnLYK3*, *BnLYK4*, and *BnLYK5* as well.

### 3.6. Expression Analysis of BnLysM-RLK Genes at Various Tissues under Biotic and Abiotic Stresses

**Figure 4:**
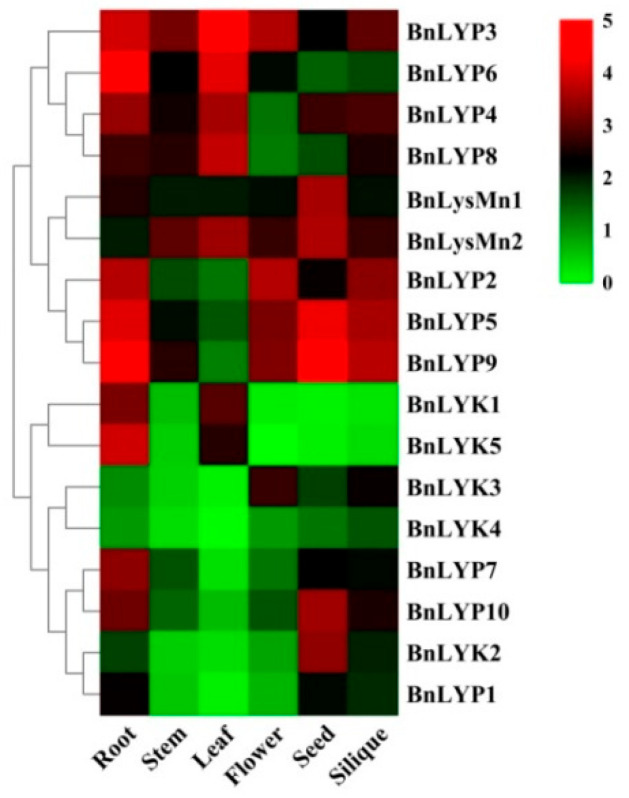
The expression pattern of *LysM-RLK* genes in different tissues. The color boxes indicate expression values, the lowest (green), medium (black), and the highest (red).

Because of its high content of unsaturated fatty acids and proteins, *B. napus* is considered one of the plants that produce the healthiest oils. Due to its outstanding properties, such as rapid growth, this plant is also used as a useful species for genetic and molecular studies of development and adaptation to diverse conditions. Therefore, in the current study, the expression of *LysM-RLK* genes has been investigated in *B.napus*. RNA-seq data sets for *B. napus* at different developmental stages tissues have been studied in leaf, flower, root, seed, stem, and silique to discover the related *LysM-RLKs* (Figure 4, Supplementary Materials: Table S6).

**Figure 5:**
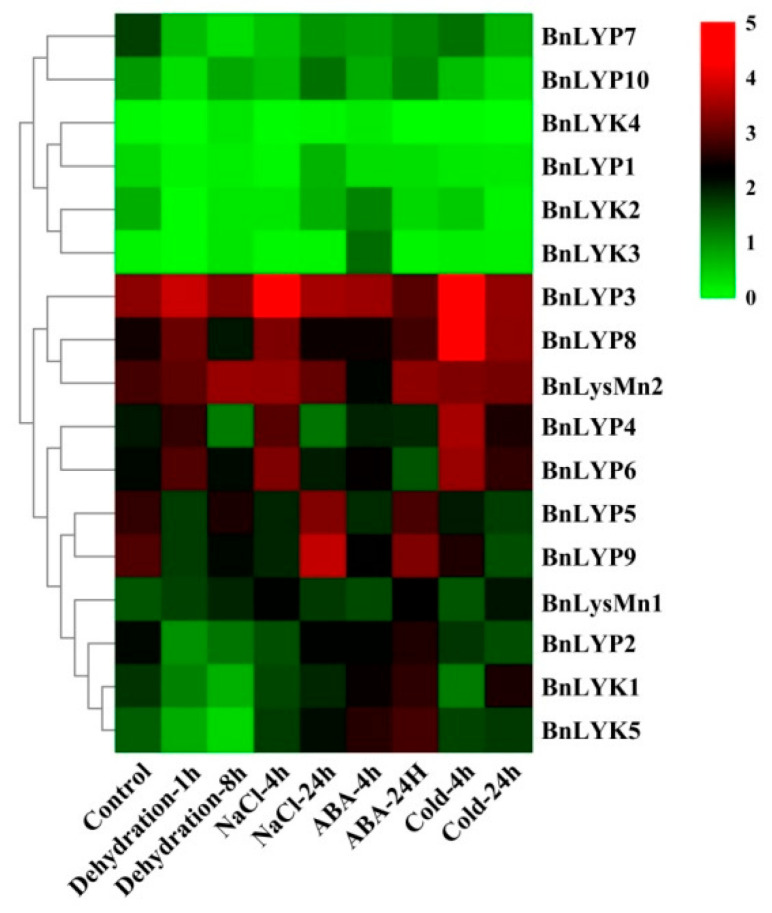
The expression pattern of *LysM-RLK* genes under abiotic stresses. The color boxes indicate expression values, the lowest (green), medium (black), and the highest (red).

Different expression patterns have been observed in members of the *LysM-RLK* family. All members of the LYP subfamily revealed moderate to high transcript levels at all developmental stages and tissues except *BnLYP1* (low expression in all tissues except seed), *BnLYP2* (low expression in stem and leaf), *BnLYP6* (low expression in seed and silique), *BnLYP7* and *BnLYP10* (low expression in the stem, leaf, and flower), *BnLYP8* (low expression in flower and seed), and *BnLYP9* ((low expression in leaf) and LYP4 low expression in flower. The highest expression in this subfamily was related to seed (*BnLYP8*, followed by *BnLYP5*), leaf (*BnLYP3*, followed by *BnLYP6*), silique (*BnLYP9*, followed by *BnLYP5*), flower (*BnLYP2*, followed by *BnLYP3*), and stem (*BnLYP3*). In the LYK subfamily, all *BnLYKs* demonstrated low expression except *BnLYK1* and *BnLYK5* (high level of transcripts in root and moderated expression in leaf), *BnLYK2* (high level of transcripts in seed), and *BnLYK3* (moderated expression in flower and silique). However, *BnLYK5* showed no obvious expression in the flower. Based on RNA-seq data analysis of the *BnLysMn* subfamily, all members demonstrated moderate to high levels of transcripts in tissues. The expression patterns of *BnLysM-RLK* genes have been examined to predict their role in responding to abiotic stresses as well (Figure 5, Supplementary Materials: Table S7).

**Figure 6:**
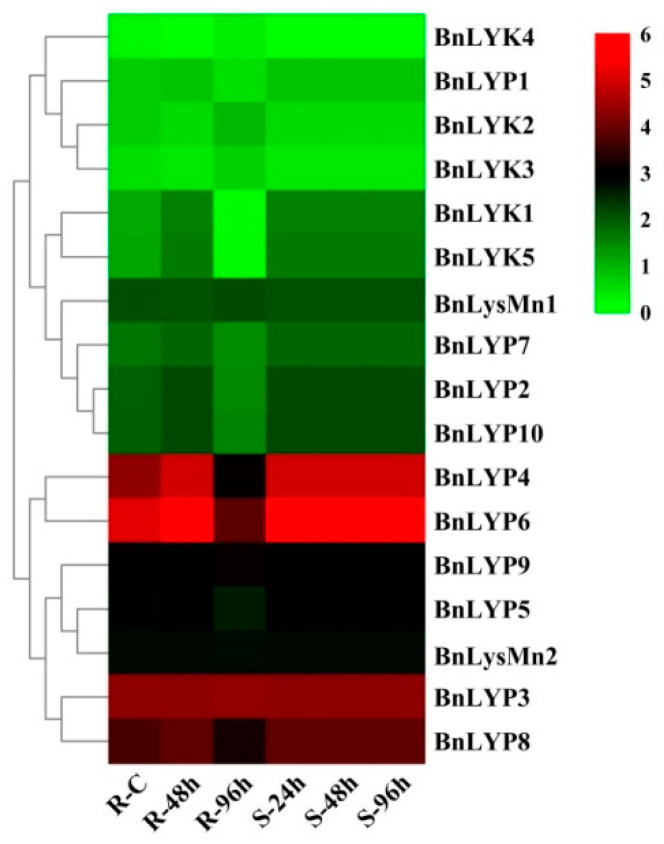
The expression pattern of *LysM-RLK* genes in response to *Sclerotinia clerotiorum* infection. The color boxes indicate expression values, the lowest (green), medium (black), and the highest (red). R, S, and C indicate resistant (J964), susceptible (J902), and control plants, respectively.

**Figure 7:**
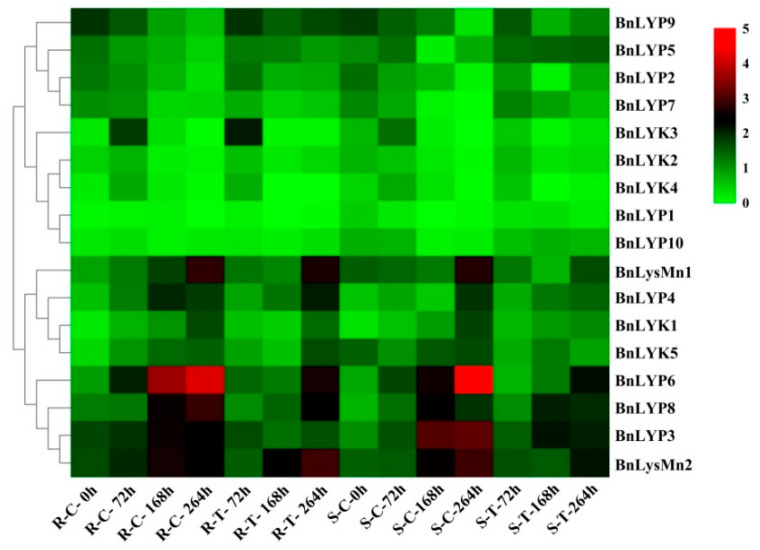
The expression pattern of *LysM-RLK* genes in response to *Leptosphaeria maculans* infection. The color boxes indicate expression values, the lowest (green), medium (black), and the highest (red). R, S, C, and T indicate resistant (DF78), susceptible (Westar), control, and treatment plants, respectively.

In response to dehydration after one hour, the down-regulated expression has been observed in *all BnLysM-RLKs* while the expression of *BnLYP3-4, BnLYP6, BnLYP8*, and *BnLYsMn1-2* was up-regulated. After 8 h of dehydration, the expression of all *BnLysM-RLKs* has been down-regulated obviously except *BnLYK3-4*, and *BnLysMn1-2* which showed up-regulation. The expression of BnLysMn1-2 could be up-regulated by all the studied stresses except *BnLysMn1* and *BnLysMn2* with no obvious and down-regulated expression in response to cold and ABA after four hours, respectively. Under NaCl treatment, the expression of *BnLYK2-3, BnLYP4,* and *BnLYP6-8* has been decreased whereas the expression of other *BnLysM-RLKs* has been induced more significantly at 24 h. The expression of *BnLYP1* and *BnLYP7* has been suppressed by all the studied stresses except *BnLYP1* showed up-regulation in response to NaCl after 24 h. The expression of 9 *BnLysM-RLKs* genes has been up-regulated under ABA stress after four hours including *BnLYk1-5, BnLYP2-3, BnLYP6,* and *BnLysMn1* while the transcript level of the *BnLYK2-4*, *BnLYP1*, *BnLYP3-4*, and *BnLYP6-7* was down-regulated after 24 h of ABA treatment. After 24 h of cold stress, the expression of *BnLYK2, BnLYK4, BnLYP1-2, BnLYP5, BnLYP7,* and *BnLYP9-10* genes has been down-regulated. The RNA-seq data sets were applied for analyzing the expression of *BnLysM-RLKs* in response to fungal pathogens including *Leptosphaeria maculans* and *Sclerotinia sclerotiorum*. In response to *S. sclerotiorum*, *BnLYP3-6*, *BnLYP8-9*, and *BnLysMn2* revealed moderate to high expression in resistance, sensitivity, and control *B. napus* whereas the lowest expression was related to *BnLYK4*. As illustrated in Figure 6, *BnLYP3-4* and *BnLYP6* are consistently highly expressed in response to *S. sclerotiorum*, showing that these genes are likely involved in response to a fungal pathogen. The expression of all *BnLysM-RLKs* revealed down-regulated expression after *L. maculans* infection while *BnLY5* and *BnLY9* showed up-regulation (Figure 7). Similarly, *BnLYP7* and *BnLYK2* have been up-regulated in both susceptible and resistant cultivars except in resistant *B. napus* after 72 h of infection. *BnLYP6* has been suppressed by *L. maculans* infection in both susceptible and resistant cultivars. In general, the expression of *BnLysM-RLKs* in response to *S. sclerotiorum* infection was much higher than the response to *L. maculans* infection (Supplementary Materials: Tables S8 and S9).

### 3.7. BnLYP6 Structural Modeling and Docking Studies

**Figure 8:**
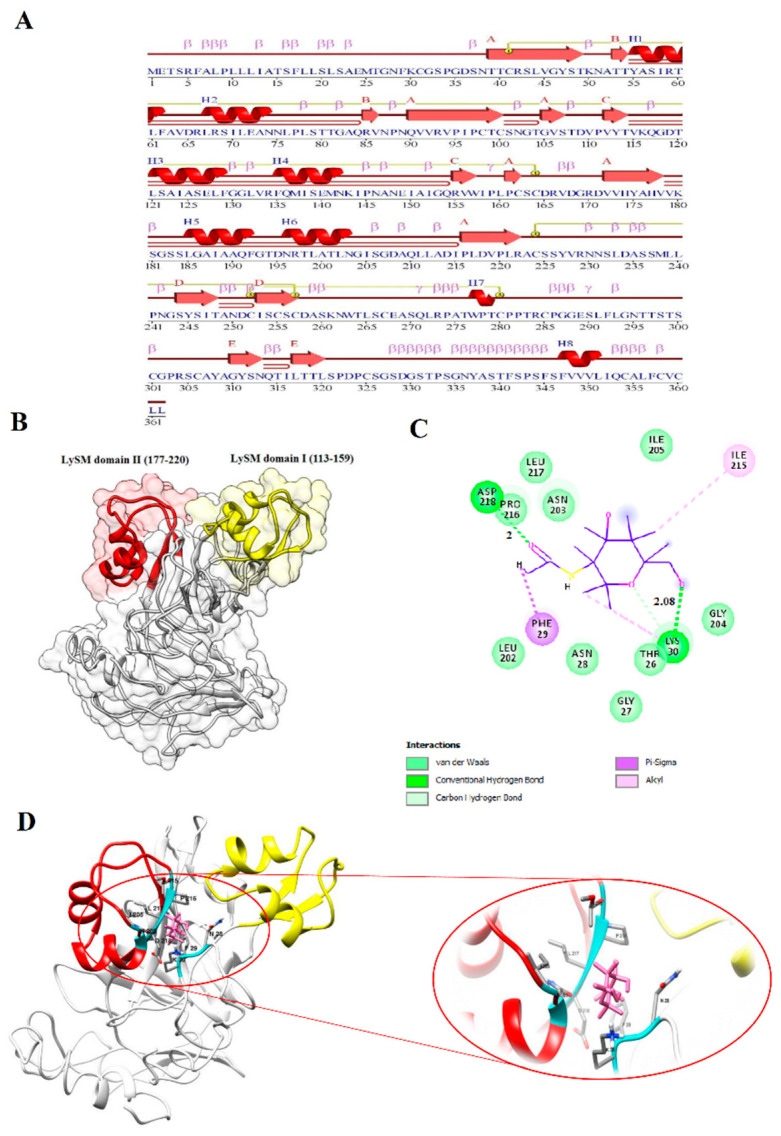
The BnLYP6 protein features. Secondary and three-dimensional model structure (**A**,**B**). The residues involved in the BnLYP6-chitin interaction (**C**). Docking studies of the three-dimensional structure of chitin onto the predicted model of BnLYP6 (**D**). AutoDock v4.2.6 has been used to analyze ligand-protein interaction.

In the current investigation, the highest expression in response to biotic stress was related to *BnLYP6*, thus, its molecular structure and ligand-enzyme interaction were investigated. Because PGN and chitin are structurally similar, LYP4 and LYP6 may also physically bind to chitin [75]. I-TASSER and ModRefinder servers have been used to predict and refine three-dimensional structures of BnLYP6 protein. Based on the results of the Ramachandran analysis of non-refined and refined models, the residue count increased in favored regions from 63.7% to 78.1%, which indicates the efficiency of the refinement stage and increase the quality of the modeled structure (Supplementary Materials, Table S10 and Figure S1). The modeled structure for BnLYP6 revealed 8 helices, 14 strands, 6 beta hairpins, 71 beta turns, and 4 gamma turns (Figure 8A). The BnLYP6 structure contains two domains, including the LysM domain I (residues 113–159) and the LysM domain II (residues 177–220) (Figure 8B). The LysM domain is varied in size, ranging from 35 to 50 amino acids. LysM domain I and II revealed three-dimensional βααβ structure, which is inconsistent with the structure of LysM domains in other studies, implying that this structure is highly conserved [78].

Docking analyses of chitin on the refined model structure were performed using AutoDock 4.2 to investigate the ligand specificity of *B. napus* LYP6. According to docking simulation with the ligand-enzyme binding energy of -7.9 kcal/mol, THR26, GLY27, ASN28, PHE29, LYS30, LEU202, ASN203, GLU204, ILE215, PRO216, LEU217, and ASP218 6 formed closed contacts with the docked chitin (Figure 8C,D). The chitin formed a hydrogen band with ASP218 and LYS30 of BnLYP6. Hydrogen bonds are the most significant weak interactions in biology. The ligand-enzyme complex seems to be more stable due to a large number of intermolecular hydrogen bonds [79]. In the current study, two hydrogen bonds have been observed between chitin and BnLYP6. On the other hand, the shorter the hydrogen bond, the stronger the bond and the more stable structure. Therefore, the interaction between BnLYP6 and chitin is stable due to the existence of two hydrogen bonds with a length of about 2 Å.

### 3.8. The Codon Usage Bias Analysis of Brassica LysM-RLK

The results of the codon usage bias analysis have been shown in Supplementary Materials (Supplementary Materials, Table S11). The GC value for *Brassica LysM-RLK* genes was between 0.437 and 0.548, while the GC3s value was between 0.383 and 0.617. Because of the strong correlation between GC and GC3, the mutation is the most important factor in codon creation (Table 3).

**Figure 9:**
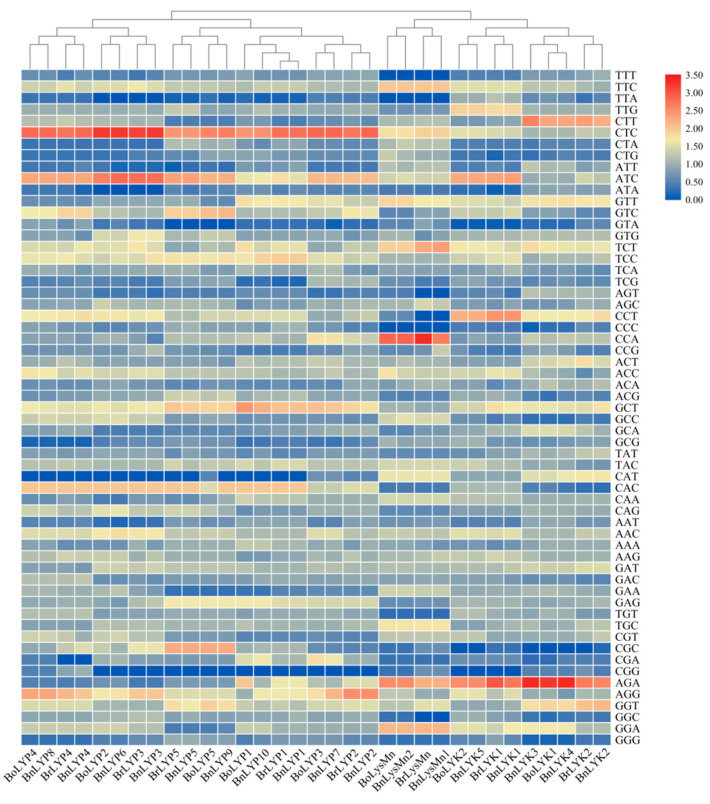
Relative synonymous codon usage analysis (RSCU) values of *Brassica* LysM-RLKs are represented as a heat map. The color boxes represent RSCU values, with the lowest (blue) and maximum (red) codon usage. TBtools was used to construct the heatmap.

The CAI (codon adaptation index), which was in the range of 0.221–0.262 in *Brassica LysM-RLKs*, is typically used to predict gene expression levels. The closer CAI is to 1, the stronger the codon preference and the higher the gene expression. A relative synonymous codon usage (RSCU) > 1 implies that codons are used more frequently than other synonymous, an RSCU = 1 indicates that codons are not preferred, and an RSCU of 1 indicates that codons are rarely utilized by genes [80]. There are 21 codons in *BnLYP7*, 22 *BnLYP9*, 23 codons in *BoLYP3-5*, *BnLYP4-5*, and *BnLYK4*, 24 codons in *BrLYP4-5* and *BnLYP2*, 25 codons in *BoLYK1-2*, *BoLYP1*, *BrLYP2*, *BrLYK1*, *BnLYP5*, and *BnLYK5*, 26 codons in *BnLYK3*, 27 codons in *BrLYP3*, *BrLysMn*, and BnLYK1, 28 codons in *BoLYP2*, *BnLYK2*, *BnLYP1*, *BnLysMn1*, and *BnLYP3*, 29 codons in *BrLYP1* and *BnLYP6*, and 30 codons in *BoLysMn*, *BrLYK2*, *BnLysMn2*, and *BnLYP1* with RSCU > 1 indicating that these are the most desired codons for each gene. The higher RSCU value (the more frequent codons for each gene) is shown in red, while the lower RSCU value is shown in blue (Figure 9). According to the RSCU value, *Brassica LysM-RLKs* were divided into four clusters: cluster I (*BnLYK2-3, BoLYK1-2, BrLYK1,* and *BnLYK4-5*), cluster II (*BnLysMn1-2, BrLysMn,* and *BoLysMn*), cluster III (*BrLyP1-2, BnLYP1-2, BoLYP1, BoLYP3, BoLYP5, BrLYP5, BnLYP5*, and *BnLYP7*), and cluster IV (*BoLYP2, BnLYP3-4, BrLYP3-4, BoLYP4, BnLYP6*, and *BnLYP8*). Each cluster had a similar preference for codons.

## 4. Discussion

LysM-RLKs play an important role in the plant immune system against pathogens [17]. In the current study, 33 *LysM-RLK* genes were found among three *Brassica* species. In *B. napus*, 17 genes were detected, while only 8 genes were identified in each of *B. oleracea,* and *B. rapa*. Study of RGAs of 30 species of Brassicaceae showed that between 5 and 14 *LysM-RLK* genes are present in the different assembly versions of *B. napus* [4]. The study of RLK and RLP in *Brassica juncea* also showed that the number of *LysM-RLK* genes in this plant is low and in contrast, *LRR-RLK* genes have a high frequency [4,81].The observed difference in the number of *LysM-RLK* genes identified in this study may be due to differences in detection criteria and differences in *B. napus* assembly versions. RLKs has been identified in many plants such as *Arabidopsis thaliana*, *Oryza sativa*, *Brachypodium distachyon*, *Citrus sinensis*, *Triticum aestivum*, *Gossypium hirsutum*, *Pyrus bretschneideri*, *Malus domestica, Solanum tuberosum* and *B. juncea* that containing 14, 20, 11, 9, 117, 60, 18, 21, 10 and 11 RLKs genes, respectively [16,17,39,40,43,78,81,82]. Due to the variability of the number of genes in different plant species, it can be concluded that the expansion of the *LysM-RLK* gene family is species-specific resulted from gene duplication events [83]. Based on the number of detected *LysM-RLKs*, it may be concluded that there is no meaningful association between genome size and the number of genes in plants. For instance, *Triticum aestivum* and *Gossypium hirsutum* each have 117 and 60 *LysM-RLK* genes, while their genome sizes are 17 Gb and 2.5 Gb, respectively. The identified *Brassica LysM-RLK* were categorized into LYK, LYP, and LysMe groups. The distribution of *LysM-RLK* was uneven *Brassica* genome. Most, if not all, flowering plants had one or more genome duplication events in their evolution [84]. In the current study, only segmental duplication resulted in multiple copies of *LysM-RLK* genes in *Brassica*.

The Ka/Ks ratios of the most duplicated *LysM-RLKs* were less than 1 except for three duplicated gene pairs (*BnLysMn2/BnLysMn1*, *BnLysMn2/BrLysMn*, and *BnLYP6/BrLYP3*) with Ka/Ks more than 1 and two duplicated gene pairs (*BnLYP7/BoLYP3* and *BnLYP8/BoLYP4*) with no Ka/Ks value due to the same sequence. It should be noted that during evolution, changes in the coding region of duplicated genes resulted in various functions due to amino acid substitution or exon-intron structural divergence [85]. Because of the high purifying selection in the *LysM-RLK* gene family, the importance of the functional role of *Brassica LysM-RLK* genes has been determined. According to the phylogenetic tree, it was shown that *Brassica* LysM-RLKs have a close relationship with their counterparts due to their sequence conservation and similar function. The amino acid compositions of each cluster were similar, implying that the phylogenetic distribution of *Brassica* LysM-RLK proteins is associated with their motif contents. All members of the LYK cluster contained 10 common motifs 1, 3-4, 7-8, and 11-15. The difference between the members of this subfamily was related to motif 5 in the clade of BoLYK1, BnLYK2, BrLYK2, BnLYK3, and BnLYK4, while the clade of BnLYK1, BrLYK1, BoLYK2, and BnLYK5 contained motif 2. These results are completely consistent with the results of the phylogenetic tree. The LysMn subfamily had common motifs 1, 4, 8, 12, and 13. In the LYP subfamily, three groups were observed. The first group (Cluster I) consisted of BrLYP2, BnLYP2, BoLYP3, BoLYP5, BrLYP, BnLYP5, BnLYP7, and BnLYP9 proteins with common motifs 1-7 and 9-12. The second group (Cluster II), including BnLYP1, BoLYP1, BrLYP1, BnLYP10 with common motifs 1-7, 9-12, and 14 except for BoLYP1 with extra specific motif 13. The third group (Cluster III) consisted of BoLYP2, BrLYP3, BnLYP3, BnLYP4, BrLYP4, BoLYP4, BnLYP6, and BnLYP8 demonstrated 11 same motifs 1-7 and 10-13. The difference between clusters I and II was related to the existence of motifs 14 in the second cluster while cluster III was separated from the above two clusters due to the lack of motif 9. The structure of exons and introns, as well as the splicing phase, play crucial roles in the evolution of gene families [86]. The high and highest conservations were found in intron phases 0 and 1, respectively, while the lowest conservation was found in intron phase 2 [87,88]. The frequency of phases 0 and 1 in all subfamilies was higher than in phase 2, including LYK (63.63%), LYP (55%), and LysMn (100%) indicating high conservation of protein function during *Brassica* evolution. 9.09% of *Brassica LysM-RLK* genes were intronless.

The study of promoter regions is necessary to understand the function of *Brassica LysM-RLK* genes. In response to environmental stresses, transcription factors play a significant role. They bind to the target genes’ promoters, regulating their expression [89]. The presence of regulatory components related to stress, developmental stage, light, and phytohormones suggests that *LysM-RLKs* have a role in the plant’s response to a variety of biological processes. Several cis-elements associated to plant resistance against biotic and abiotic stresses were identified based on the promoter analysis, including ARE, DRE, GC-motif, LTR, MBS, MYB, MYC, STRE, AP1, S-box, W-box, WUN-motif, and WRE3. The TGACG and CGTCA motifs are found on methyl jasmonate-responsive genes [90]. Senescence, seed germination, and response to biotic and abiotic stressors are all affected by jasmonate as well [91]. In response to ABA, the ABRE, ABRE3a, and ABRE4 motifs are activated, resulting in drought and salinity tolerance in plants. The high frequency of cis-acting elements associated with response to drought, pathogen, cold, ABA, auxin, jasmonate, gibberellin, and ethylene suggests that *LysM-RLK* genes are active in a variety of stress responses in *Brassica* species. However, the existence of specific regulatory elements is not sufficient evidence for these genes’ responses to specific hormones or stresses, requiring the use of laboratory procedures to precisely determine their function. SSRs are 1-6 nucleotide tandem repeats that have been shown to play a crucial function in gene regulation [92]. In the current study, tetra-nucleotide repeats (40.91%) were found to be more common than other SSRs. The type of dominant SSRs varies in various plant species, and the abundance of AT repeats is higher in the dicots genome than monocots [93]. SSR polymorphisms in *LysM-RLK* may be examined in different cultivars in the future, and they may be useful for marker-assisted selection (MAS) development in *Brassica* genetic improvement to choose genotypes with higher resistance to various stresses. MicroRNAs (miRNA) are non-coding small RNAs with a length of 19-24 bp. They are crucial in the regulation of post-transcriptional modifications. Plants, animals, and viruses all have miRNAs. Plant development and responses to environmental stressors are also influenced by them [94]. *Brassica* miRNAs targeted 6, 5, and 1 transcript in the LYK (BnLYK1, BrLYK1, BoLYK1, BnLYK3-5), LYP (BnLYP2-3 and BrLYP2-3), and LysMn (BnLysMn2) subfamilies, respectively. No *LysM-RLK*-targeted miRNA was found in LysMn and LYP subfamilies. miR156 is required for the vegetative phase transition of a plant from a juvenile to an adult [95]. Under normal growth conditions, auxin-induced miR390 stimulates lateral root development [96]. Therefore, *BnLYK1* and *BnLYK3-5* are likely to play a role in root development. miR396 with reduced activity has been demonstrated to give widespread resistance to necrotrophic and hemibiotrophic fungal infections in Arabidopsis [97], thus, BnLysMn2 may be involved in the *B.napus* defense against fungal infections. miR397 has been reported that target laccase family genes through transcript cleavage in Arabidopsis and rice [98]. As a result, they are required for the maintenance of cell walls and vascular integrity, implying that they play a role in plant defense against various stresses [99]. In Arabidopsis, banana, and rice, miR397 has been shown to have a major impact on plant biomass and yield [99,100,101] that targets *BnLYK4* in this study. miR5717 regulates genes involved in lipid metabolism and pollen tube growth [102]. Therefore, *BrLYP3* is likely to have a role in reproductive development. It was hypothesized that miR5721 may target genes that encode biotinyl-lipoyl-containing proteins [103]. In *B. napus*, miR2111 plays a significant role in the response to phosphorus deficiency [104]. Finally, miR6029 has been reported to regulate fatty acid production during the development of *B. napus* seeds [105].

The expression profile of genes provides important information about the function of the genes that have been found. According to recent studies, *RLKs* are thought to play a crucial role in stress responses [106,107]. The highest number of *BnLysM-RLK* genes with moderate to high expression was observed in seeds (76.92%) followed by roots (76.47%), and silique (52.94%) while the lowest number of moderate to highly expressed genes was related to stem (35.39%) preceded by leaf and flower (41.17% each). *BnLYK5* was considered not expressed in flower tissue. The highest expression in root and flower tissues was related to *BnLYP6* and *BnLYP2*, respectively, while in stem and leaves the highest expression was related to *BnLYP3* and in seed and silique was related to *BnLYP9*. Most of the low-expression and high-expression genes were related to LYK and LYP subfamilies, respectively. The results demonstrated that the expression patterns of genes belonging to the same subfamily can differ significantly. For instance, the *BnLYP3* and *BnLYP8* of the LYP subfamily are consistently expressed at high levels while other *LYP* genes demonstrated a minimum expression except for *BnLYP4-6* and *BnLYP9* with moderate to high expressions. The results reinforced the hypothesis of divergence that the duplicated genes may be the result of one of two processes: 1) subfunctionalization, and 2) neofunctionalization. In the subfunctionalization process, some of the characteristics of new genes vary from the parental genes [108], whereas the new gene plays a different role in the neofunctionalization process due to differences in amino acid content [109]. Drought is one of the important environmental stresses that have negative effects on plant growth. RLKs’ response to drought stress is influenced by ABA [107]. ABA is a key plant hormone, regulating the expression of genes involved in drought, salt, and osmotic stress responses [110]. As an ABA-dependent pathway, *Arabidopsis* receptor dead kinase 1 (RDK1) plays an important role in drought stress response. The *Arabidopsis* rdk1 mutants were hypersensitive to drought stress due to the down-regulation of ABA-responsive genes [111]. Considering the present study, the expression of *LysM-RLK* genes in response to abiotic stresses varies depending on the stress type and duration. Thus, *BnLYP3* and *BnLYP8* genes were up-regulated by salt after 4 h of treatment, while they were down-regulated after 24 h under salinity condition. The highest transcript level under dehydration conditions after 1 and 8 h was related to *BnLYP3* and *BnLysMN2*, respectively. Interestingly, in all treatments including salt (after 4 h), ABA (after 4 h), and cold (after 4 and 24 h) *BnLYP3* showed the highest expression while the transcript levels of *BnLYP9* and *BnLysMn2* was higher than other *LysM-RLKs* in response to salinity and ABA treatments after 24 h, respectively. These findings suggested that the *BnLYP3* gene may play a critical role in *B. napus* response to abiotic stresses, which can be utilized to improve the resistance of *B. napus* cultivars in future researches. We can also suggest this gene as a marker of abiotic stresses in *B. napus.* Pathogens and pests are believed to be capable of causing 50–60% losses in Brassica crop yield and quality, resulting in significant economic losses [112]. Sclerotinia stem rot is one of the most destructive diseases for *B. napus*, caused by *S. sclerotiorum*. The highest expression in response to *S. sclerotiorum* was related to *BnLYP6*, followed by *BnLYP4*, *BnLYP3*, *BnLYP8*, and *BnLYP5* in both susceptible and resistant cultivars except in resistant cultivar after 96 h that the highest expression was related to *BnLYP3*, followed by *BnLYP6*, *BnLYP8*, *BnlYP9*, and *BnLYP4*. Based on the results of Brotman et al. (2012), CERK1 (LysM-RLK1) receptor is required for chitinase-induced salt and heavy metal tolerance in plants. Likewise, they suggested that ectopic chitinases are largely involved in inducing plant immune response against pathogens mediated by the CERK1 receptor [26]. June et al. (2015) revealed that GbRLK plays an important role in modulating a variety of plant-pathogen interactions in *Gossypium barbadense*. According to their findings, the majority of the up-regulated genes associated with disease resistance were chitin responsive, implying that the transgenic Arabidopsis showed improved resistance against *Verticillium dahlia* by modulating the chitin response signaling pathway [113]. Blackleg disease, caused by *L. maculans*, is a serious production limitation in *B. napus*. It has been observed in all canola-growing regions except China and causes yearly yield losses of 10–20% [114]. Expression in *BnLysM-RLKs* is suppressed after *L. maculans* infection, except in *BnLYP5* and *BnLYP9* that their expression was slightly increased after pathogen infection. Taken collectively, all members of the gene family are expressed in *B. napus*.

The study of the *LysM-RLK* gene family in other plants also shows the response of these genes to fungal and bacterial pathogens. A study of transcriptome data has shown that the expression of wheat *LysM-RLK* genes is induced in response to Flg22 and chitin. Therefore, these genes are involved in wheat resistance to fungal and bacterial pathogens [40]. In *Citrus sinensis*, the expression of *LYK* genes have increased in response to *Xanthomonas citri*, the Citrus bacterial canker (CBC) causing plant bacterial pathogen, and the salicylic acid (SA), methyl jasmonate (MeJA), and abscisic acid (ABA) hormones. Accordingly, there is a link between the *LYK* genes, the ABA, SA, and MeJA signaling pathways, and CBC resistance [17]. *Fusarium graminearum* (Fg), the causative agent of *Fusarium* head blight (FHB), induces the expression of *BdLYK2*, *BdLYK3,* and *BdLYK4* genes in *Brachypodium distachyon*. On the other hand, the expression of *BdLYP1* and *BdLYP4* genes has decreased in response to this pathogen. The function of these genes seems to be similar to the Arabidopsis *AtLYP2* and *AtLYP3* genes, which are involved in responding to bacterial pathogens [78]. Although these results can confirm the specificity of *LYP* genes to bacterial PGN, in rice, *LYP4* and *LYP6* genes are dual-functional and can respond simultaneously to fungal chitin and PGN [115]. The present study also showed that *BnLYP6* gene expression is induced in response to fungal pathogens. On the other hand, molecular docking analysis showed that *BnLYP6* has a high affinity for chitin, which indicates the role of this gene in responding to fungal pathogens in *B. napus*. These results together indicate the different functions of *LysM-RLK* genes in the response of plants to bacterial and fungal pathogens as well as abiotic stresses [116].

CUB can represent the origin of a gene and can be utilized as a theoretical model for analyzing gene evolution and function [117]. The amount of ENC varies between 20 to 61, and the higher the ENC value, the weaker the CUB. The ENC of the *Brassica* LysM-RLKs ranged from 48.97 to 59.64, indicating that the codons of this family are not affected by strong codon bias and there are various synonymous codons [118]. The CAI index varies from 0 to 1, which is typically applied to measure expression levels [119]. According to the CAI index (0.221-0.2621), the expression efficiency of *the BnLysM-RLKs* is almost low. Although the codon preference of highly expressed genes is stronger with a higher CAI and lower NC values, low-expression genes have more rare codons, resulting in a lower CAI and a higher NC. For instance, *BnLYP6* showed increased expression in response to biotic stresses with almost larger CAI and relatively lower NC. The optimal codon frequency is represented by the FOP and CBI indices, which range from 0 to 1 and -1 to 1, respectively. Based on the results of the FOP and CBI, the frequency of optimum codons in this gene family was low. The majority of *Brassica* LysM-RLKs showed a GC content of more than 0.5, implying that *Brassica* LysM-RLKs have obvious preference for GC. 69.69% of *Brassica* LysM-RLKs demonstrated a GC3s value greater than 0.5, indicating that G/C end codons are preferred.

## 5. Conclusions

Bioinformatic analyses were performed in this work to discover 33 *LysM-RLK* genes with significant structural diversity in three *Brassica* species. Based on the phylogenetic analysis, *Brassica LysM-RLK* genes were divided into three groups including LYK, LYP, and LysMn. Only segmental duplication was found during the investigation of the mechanism of gene family expansion. The function of most duplicated *Brassica LysM-RLK* genes has been conserved over evolution due to negative selection. During promoter analysis, several elements in the *Brassica LysM-RLK* promoters were found, showing that they play a role in stress response and plant growth. 22 SSR and 39 miRNA were detected which can be employed in MAS and genetic transformation, respectively. The functional involvement of *LysM-RLK* genes in *Brassica* tissues in response to environmental stressors was revealed by their expression patterns in diverse tissues. Due to the high expression of *BnLYP3* genes in response to *Sclerotinia* stem rot infection and *BnLYP3* in response to abiotic stresses, these genes can be exploited in the production of *B. napus* plants resistant to biotic and abiotic stresses. The discovery of these residues might be important in future investigations to improve the efficiency of the LYP6 enzymes and generate pathogen-resistant *B. napus* by site-directed mutagenesis. This research has given fundamental information on the *LysM-RLK* genes, which will be useful in future investigations aimed at improving Brassica quality.

## Figures and Tables

**Table 1:** Features of Brassica LysM-RLK proteins.

Gene Name	Gene Stable ID	Chromosome /Scaffold Name	Gene Start (bp)	Gene End (bp)	Strand	Length	Weight (kDa)	pI	Localozation
BnLYK1	GSBRNA2T00068250001	A4	11744720	11746625	−1	602	65.77	5.32	PlasmaMembrane
BnLYK2	GSBRNA2T00021233001	A6	1183129	1186599	1	633	69.26	5.95	PlasmaMembrane
BnLYK3	GSBRNA2T00085753001	A8	1474443	1478188	1	665	72.76	5.74	PlasmaMembrane
BnLYK4	GSBRNA2T00010694001	C3	6266494	6270198	−1	664	72.61	5.84	PlasmaMembrane
BnLYK5	GSBRNA2T00149947001	C4	37709852	37711907	−1	598	65.24	5.41	PlasmaMembrane
BnLYP1	GSBRNA2T00065733001	A2	11064901	11067027	−1	422	43.95	5.12	Extracellular
BnLYP2	GSBRNA2T00125469001	A6	8524836	8526824	1	414	43.22	4.83	PlasmaMembrane
BnLYP3	GSBRNA2T00147404001	A6	17944793	17946593	−1	362	38.19	7.78	Extracellular
BnLYP4	GSBRNA2T00047588001	A7	2800269	2802134	−1	364	38.76	7.31	Extracellular
BnLYP5	GSBRNA2T00102765001	A8	15737129	15739025	−1	415	43.35	4.64	PlasmaMembrane
BnLYP6	GSBRNA2T00125046001	C3	32553799	32555600	1	362	38.17	6.12	Extracellular
BnLYP7	GSBRNA2T00119287001	C5	10798534	10800145	1	416	43.47	4.83	Extracellular
BnLYP8	GSBRNA2T00145018001	C7	9946891	9949154	1	364	38.83	7.77	Extracellular
BnLYP9	GSBRNA2T00066647001	C8	22779362	22781576	1	414	43.35	4.64	PlasmaMembrane
BnLYP10	GSBRNA2T00080311001	Cnn	10720216	10722392	−1	422	4.08	5.12	Extracellular
BnLysMn1	GSBRNA2T00081535001	Ann	7838524	7839838	−1	260	28.92	6.22	Extracellular
BnLysMn2	GSBRNA2T00148567001	C4	16939318	16941025	1	260	28.91	6.22	PlasmaMembrane
BoLYK1	Bo3g181300	C3	62973513	62976832	1	665	72.66	5.76	PlasmaMembrane
BoLYK2	Bo4g151880	C4	41862968	41864776	−1	602	65.6	5.32	PlasmaMembrane
BoLYP1	Bo2g092750	C2	25211400	25213688	1	475	5	6.7	Extracellular
BoLYP2	Bo3g092250	C3	33860217	33861898	1	362	38.27	6.13	Extracellular
BoLYP3	Bo5g034850	C5	11423329	11424940	1	416	43.45	4.83	Extracellular
BoLYP4	Bo7g014960	C7	5770672	5773498	−1	364	38.86	7.77	Extracellular
BoLYP5	Bo8g071130	C8	23528006	23530169	1	414	43.31	4.72	PlasmaMembrane
BoLysMn	Bo4g078290	C4	17241540	17243011	−1	260	28.92	6.22	PlasmaMembrane
BrLYK1	Bra032146	A4	10968263	10970071	−1	602	65.84	5.4	PlasmaMembrane
BrLYK2	Bra018937	A6	1151733	1154752	1	634	69.2	6.04	PlasmaMembrane
BrLYP1	Bra008320	A2	13690581	13692699	1	422	43.95	5.12	Extracellular
BrLYP2	Bra017956	A6	8777590	8779193	1	414	43.25	4.83	Extracellular
BrLYP3	Bra009660	A6	17045973	17047648	−1	362	38.1	7.78	Extracellular
BrLYP4	Bra002021	A7	2492928	2494398	−1	364	38.84	6.68	Extracellular
BrLYP5	Bra016402	A8	17512432	17514340	−1	415	43.33	4.64	PlasmaMembrane
BrLysMn1	Bra038977	Scaffold000157	101880	103520	−1	260	28.91	6.52	Extracellular

**Table 2:** Simple sequence repeats (SSR) were detected in Brassica LysM-RLK genes.

Seq ID	Count	Motiif
BnLYP8	1	(CAAG)3
BnLYP4	1	(CAAG)3
BnLYK1	1	(CTC)4
BnLYP5	2	(CCTT)4, (TGTGG)3
BnLYP9	4	(CT)7, (CT)9, (AAG)4, (CCTT)4
BnLYP2	4	(TC)7, (TC)6, (GA)7, (AGTC)3
BrLYP4	1	(CAAG)3
BrLYP2	1	(AGTC)3
BrLYK1	1	(CTC)4
BrLYP5	1	(TGTGG)3
BrLysMn	1	(TATAT)3
BoLYP4	1	(CAAG)3
BoLYP1	1	(CT)9
BoLYK2	1	(CTC)4
BoLYP5	1	(CCTT)4

**Table 3:** The correlation coefficient between the parameters of codon usage of Brassica LysM-RLK gene family.

	CAI	CBI	Fop	ENC	GC3s
CBI	0.7 **				
Fop	0.73 **	0.99 **			
ENC	−0.12 ^ns^	−0.03 ^ns^	−0.1 ^ns^		
GC3s	0.59 **	0.81 **	0.78 **	0.18 ^ns^	
GC	0.69 **	0.88 **	0.85 **	0.25 ^ns^	0.90 **

^ns^ and ** are not-significant and significant at 1% probability level respectively.

## Data Availability

The data presented in this study are available as Supplementary Files.

## Supplementary Materials

The following supporting information can be downloaded at: https://www.mdpi.com/article/10.3390/cells11010037/s1, Figure S1: Ramachandran plot for non-refined and refined modeled structure. Table S1: Features of *Brassica* LysM-RLK proteins. Table S2: *Brassica* LysM-RLK domains. Table S3: Ka/Ks analysis of the *Brassica LysM-RLK* duplicated paired genes. Table S4: Putative conserved motifs of *Brassica* LysM-RLK. Table S5: List of Cis-acting regulatory elements in the *Brassica LysM-RLK* promoter. Table S6: Putative *Brassica* LysM-RLK- targeted miRNA. Table S7: RNA-seq data analysis of *Brassica* LysM-RLK at various tissues. Table S8: RNA-seq data analysis of *Brassica* LysM-RLK under abiotic stress. Table S9: RNA-seq data analysis of *Brassica LysM-RLK* in response to *Sclerotinia sclerotiorum* infection. Table S10: RNA-seq data analysis of *Brassica LysM-RLK* in response to *Leptosphaeria maculans* infection. Table S11: Relative synonymous codon usage analysis (RSCU) of *Brassica* LysM-RLKs.
