# Supplementary figures and images for: Analyses of Lysin-motif Receptor-like Kinase (LysM-RLK) Gene Family in Allotetraploid Brassica napus L. and Its Progenitor Species: An In Silico Study
Source: Cells. 2021 Dec 23;11(1):37. doi: 10.3390/cells11010037 (PMC8750388; doi:10.3390/cells11010037)

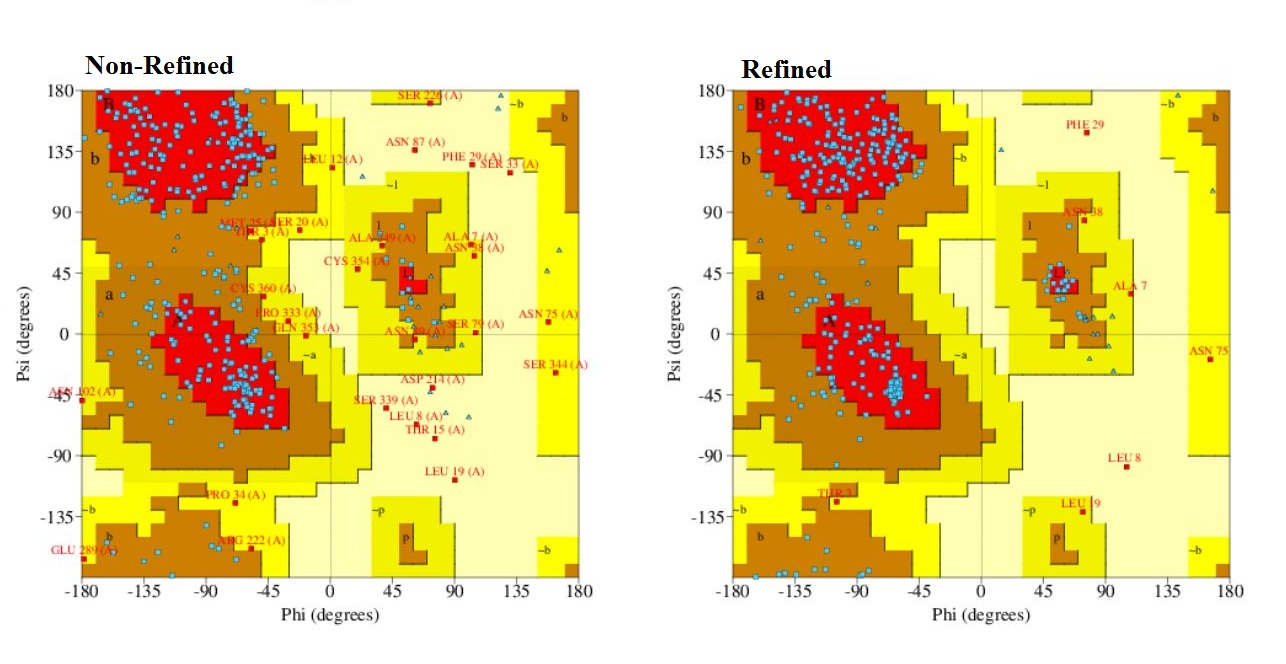

Supplement: Supplementary file 1 [file cells-11-00037-s001.zip › Figure S1.tiff]
